# Supplementary material for: A Comprehensive Review on the Role of Resting-State Functional Magnetic Resonance Imaging in Predicting Post-Stroke Motor and Sensory Outcomes
Source: Neurol Int. 2024 Jan 19;16(1):189–201. doi: 10.3390/neurolint16010012 (PMC10892788; doi:10.3390/neurolint16010012)
Supplement: Supplementary file 1 [file neurolint-16-00012-s001.zip › neurolint-2806128-supplementary.pdf]

Table S1. Search strategy and selection criteria for the most representative rs-fMRI studies in association to motor and sensory outcome after stroke which were included in the comprehensive review.

**Search strategy:**	A literature research of two databases (MEDLINE and Science Direct) was conducted by one investigator in order to trace all relevant studies published between January 1, 2012 and December 31, 2022, using either "resting state functional magnetic resonance imaging" as keyword or related term "resting state fMRI "as a search criterion. Also, the terms "stroke prognosis" or "stroke outcome" or "stroke recovery" were used as a second search criterion. The retrieved articles were also hand searched for any further potential eligible articles. Any disagreement regarding screening, or selection process, was solved by a second investigator until a consensus was reached. Supplementary Figure 1 presents the review flowchart.
**Selection criteria:**	Only full-text original articles published in the English language that focused on motor and/or sensory outcomes in stroke patients were included for further analyses. Secondary analyses, reviews, case reports, guidelines, meeting summaries, comments, unpublished abstracts, or studies conducted in animals were excluded. There was no restriction on study design or sample characteristics.
**Data extraction:**	Data extraction was performed using a predefined data form created in Excel. We recorded author, year of publication, number and age of participants, study design with regards to rs-fMRI acquisition (cross-sectional, longitudinal), type of stroke, time of rs-fMRI acquisition, main rs-fMRI parameters (MR field strength, duration of rs-fMRI acquisition), type of rs-fMRI analysis (ICA, seed-to-voxel, ROI-to-ROI analysis, etc), functional networks examined (motor, sensory, both), clinical scales utilized and main findings in terms of motor and sensory outcome and rs-fMRI networks.
**Data analysis:**	No statistical analysis or meta-analysis was performed.

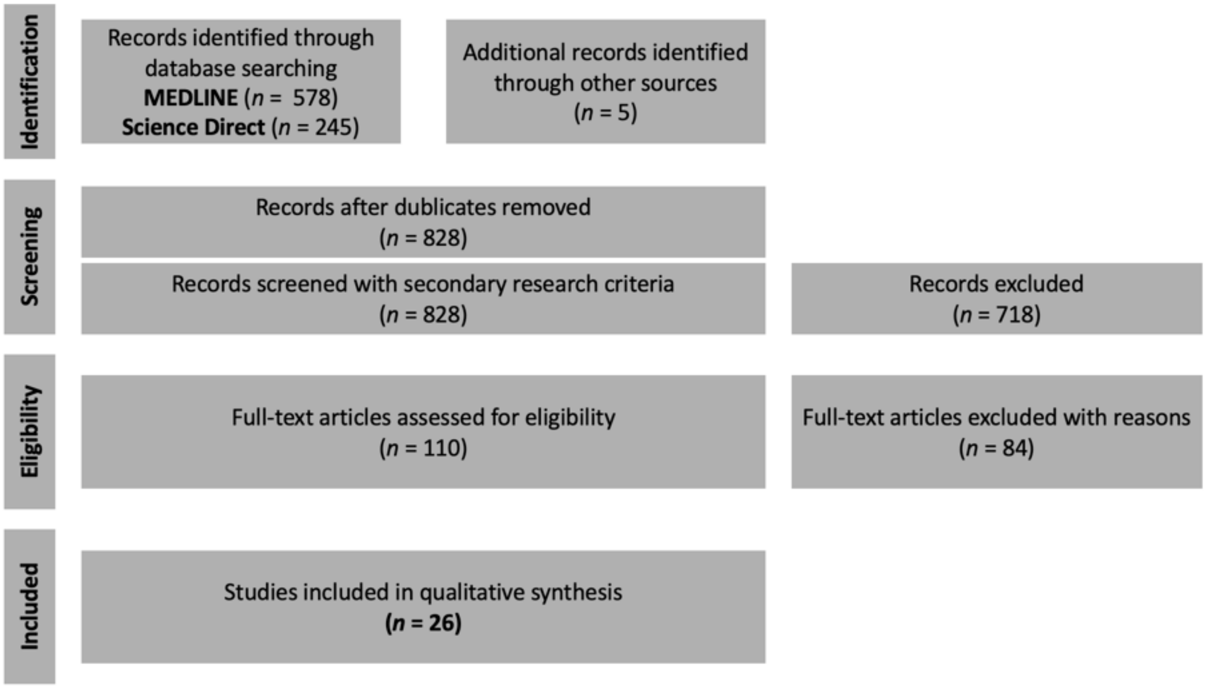

Figure S1. Study flow chart of the process of selection the most representative rs-fMRI studies in association to motor and sensory outcome after stroke.

Table S2. Representative rs-fMRI studies on motor and sensory outcome after stroke.

1st Author, (Year)	Type of stroke,<br>Study design,<br>Participants,<br>Age (years),<br>(Type of intervention for group B)	Time of rs-fMRI acquisition,<br>Field Strength,<br>Duration of rs-fMRI protocol,<br>rs-fMRI analysis,<br>Additional imaging/analysis	Main clinical, motor and/or sensory scales	Main findings
Section A: Motor outcome and rs-fMRI analysis with classic and more sophisticated approaches				
Yin, (2013) [11]	• First-ever ischemic subcortical stroke with only left motor pathway damage and normal cognitive status<br>• Cross-sectional<br>• 25 patients (PPH, *n* = 12; CPH, *n* = 12)<br>○ 61.00 ± 7.30<br>• 24 controls<br>○ 61.60 ± 9.80	• PPH: 18.80 ± 14.50 months post-stroke; CPH: 27.00 ± 25.10 months post-stroke<br>• 3.0 T<br>• 8 min 6 s<br>• ROI-to-ROI analysis within the cortical motor network, graph theoretical analysis<br>• Lesion analysis	• FMA, PHFA, MMSE	• The cortical motor-related network in both healthy controls and stroke patients exhibited small-world topology.<br>• Higher local efficiency of this network and lower global efficiency in stroke patients was found compared to healthy controls.<br>• Striking alterations in the betweenness centrality of regions were found in stroke patients, including the contralesional SMA, dorsolateral premotor cortex, and anterior inferior cerebellum.<br>• A positive correlation was found between the betweenness centrality of the contralesional SMA, contralesional anterior inferior cerebellum, ipsilesional middle frontal cortex, and ipsilesional superior parietal lobule and FMA scores.<br>• A positive correlation was found between the betweenness centrality of the bilateral dorsolateral premotor cortex and the FMA scores.<br>• No significant correlations were found between both local efficiency and global efficiency and FMA scores.
Li, (2014) [12]	• First-ever only hemispheric ischemic stroke in MCA territory<br>• Cross-sectional<br>• 24 patients (12 with dysphagia, 12 without dysphagia)<br>○ Dysphagia: 65.20 ± 4.30<br>○ Without dysphagia: 66.50 ± 5.20<br>• 12 controls<br>○ 65.80 ± 3.30	• 2–3 days post-stroke<br>• n/a<br>• n/a<br>• ROI-to-ROI analysis<br>• n/a	• P-A scale score for dysphagia	• Compared to healthy controls, stroke patients with and without dysphagia had decreased FC in the DMN and the affective network.<br>• Stroke patients with dysphagia had decreased FC in both the DMN and the affective network compared to patients without dysphagia.<br>• Dysphagia-related abnormal brain activity is not restricted to the motor network.
Lam, (2018) [13]	• First-ever stroke with unilateral upper limb deficit at chronic phase<br>• Cross-sectional<br>• 25 patients<br>○ 61.90 ± 10.20<br>• 0 controls	• 60.00 ± 69.6 months post-stroke<br>• 3.0 T<br>• n/a<br>• ROI-to-ROI analysis between L M1 and R M1 with seeds for arm/elbow and hand/finger	• ARAT, Chedoke-McMaster Stroke Assessment-Arm and Hand	• CST injury and L M1-R M1 rs-FC individually explain the variability in motor outcome.<br>• The interaction between CST injury and L M1-R M1 rs-FC was not found to significantly account for variability in motor outcome.<br>• The inclusion of both CST injury and L M1-R M1 rs-FC together significantly explains the variability in motor

		• Lesion analysis, CST injury based on T1 sequence		outcome more than either biomarker alone.
Chi, (2018) [14]	• First-ever acute unilateral stroke<br>• Cross-sectional<br>• 67 patients<br>○ Favorable outcome at 3 months, *n* = 44: median 61<br>○ Unfavorable outcome at 3 months, *n* = 23: median 59<br>• 25 controls<br>○ median 57	• 3–7 days post-stroke<br>• 3.0 T<br>• 6 min<br>• ROI-to-ROI analysis using SMN and DMN<br>• Lesion analysis, lesion maps	• NIHSS, mRS	• FC between SMN ROIs (iM1/cM1, iM1/cPCG, iM1/cPMd, iPCG/cM1, iPCG/cPCG, iPCG/cPMd, iPMv/cPMv, cM1/cPCG) was significantly lower in patients with unfavorable outcome compared to healthy controls.<br>• FC of iM1/cM1, iM1/cPCG, iPCG/cM1, and iPCG/cPMd were not different between patients with favorable outcomes and healthy controls.<br>• FC of iM1/cPMd was significantly lower in patients with unfavorable outcomes compared to patients with favorable outcomes.<br>• FC of DMN was not different between groups.<br>• FC between ipsilesional primary M1 and contralesional dorsal premotor area (PMd) was independently associated with unfavorable outcomes.
Zou, (2018) [15]	• First-ever subcortical stroke with L motor pathway damage and normal cognition<br>• Cross-sectional<br>• 36 patients<br>○ 58.75 ± 8.62<br>• 37 controls<br>○ 56.24 ± 7.87	• 16.11 ± 16.91 months post-stroke<br>• 3.0 T<br>• 8 min 6 s<br>• Graph theory analysis and topological properties of brain networks<br>• Voxel-based lesion-symptom mapping analysis	• FMA	• The data-driven approach identified 15 important sub-ROIs that mainly belong to four crucial brain areas (left precentral gyrus, right precentral gyrus, right postcentral gyrus, left postcentral gyrus) and are related to sensorimotor functions and frontoparietal control systems.<br>• The strongest functional links in healthy controls are conserved in stroke patients while the links of intermediate strength are destroyed in a non-random manner.<br>• The newly built strong connections in stroke patients result from random establishment of links across all possible brain regions.<br>• Connectivity re-organization is much more significantly expressed if a patient is severely affected by stroke. Patients with severe motor impairments (FMA <=10) experienced a greater amount of rewiring of the strong connections.<br>• Based on the voxel-based lesion-symptom mapping analysis, it appears that stroke patients do not show significant correlations between lesion location at chronic stage and both FMA scores and entropy values.<br>• Entropic measures of complexity are proposed for characterizing the functional connectivity reorganization patterns, which are correlated with hand and wrist function assessments of stroke

				patients and show high potential for clinical use.<br>• Lesion location cannot capture the functional connectivity reorganization patterns.
Hong, (2019) [16]	• Chronic left subcortical stroke<br>• Cross-sectional<br>• 52 patients<br>○ PPH, n = 26: 56.0 ± 9.92<br>○ CPH, n = 26: 56.00 ± 10.22<br>• 52 controls<br>○ 56.00 ± 8.23	• 16.11 ± 16.91 months post-stroke<br>• 3.0 T<br>• 8 min<br>• ICA and within network FC analysis	• FMA, PHFA, MMSE	• When compared to PPH patients, FC in the contralesional sensorimotor cortex within the contralesional SMN was considerably higher in CPH patients.<br>• When comparing the CHP patients to the PPH patients, there was a considerably lower FC between the ipsilesional SMN and both the dorsal SMN and ventral VN.<br>• Paretic hand performance was linked to disturbed FC patterns in large-scale brain networks in individuals with chronic left subcortical stroke.
Liu, (2015) [17]	• First-ever acute unilateral subcortical infarct<br>• Longitudinal<br>• 22 patients<br>○ Median 50.7<br>• 22 controls<br>○ Median 51	• Within week 1, 4, and 12 post-stroke<br>• 3.0 T<br>• n/a<br>• ALFF in motor-related regions (bilateral PMC, SMA, insula, precuneus)<br>• Cortical thickness of above-mentioned ROIs	• NIHSS, FMA	• Although there were no marked changes in ALFF in the bilateral PMC at week 4 compared to week 1 and healthy controls, significant increases in ALFF were found bilaterally in the PMC at week 12 compared to week 1 and healthy controls.<br>• There were no obvious ALFF changes bilaterally in the SMC, insula or precuneus during the entire follow-up period.<br>• Compared to week 1, significantly decreased mean cortical thickness were found in the ipsilesional PMC, SMC and precuneus at week 12 post-stroke.<br>• Obvious increases in mean cortical thickness in the SMC and insula were found contralesionally at week 12 compared to those at week 1.<br>• The ALFF changes in the ipsilesional and contralesional PMC were positively correlated with changes in the FM scores.<br>• The mean cortical thickness in the contralesional SMC correlated positively with changes in FM scores across all patients.
Zhao, (2018) [18]	• First-ever L subcortical stroke at chronic phase with pure motor deficits and normal cognition<br>• Cross-sectional<br>• 52 patients<br>○ CPH: 56.0 ± 10.22<br>○ PPH: 56.0 ± 9.92<br>• 52 controls<br>○ n/a	• CPH: 16.0 ± 17.36 months post-stroke; PPH: 16.0 ± 15.58 months<br>• 3.0 T<br>• 8 min 6 s<br>• ReHo measures at 3 different frequency bands<br>• Lesion analysis and lesion maps	• FMA, PHFA, MMSE	• Compared to PPH group, the CPH group showed decreased ReHo in the bilateral cerebellum posterior lobes (slow-5 band) and the contralesonal cerebellum anterior lobe (slow-4 band).<br>• Compared to PPH group, the CPH group showed increased ReHo in the contralesional SMA (slow-4 band) and the contralesional superior temporal gyrus (slow-5 band).

				• The mean ReHo values in regions with decreased ReHo were positevely correlated with the FMA scores.<br>• The mean ReHo values in regions with increased ReHo were negatively associated with the FMA scores.<br>• Significant interactions between the frequency bands and the subgroups of patients in the contralesional precentral gyrus and middle frontal gyrus.
Liang, (2020) [19]	• First-ever unilateral ischemic stroke at subacute phase<br>• Cross-sectional<br>• 20 patients<br>○ 61.10 ± 10.61<br>• 19 controls<br>○ 60.32 ± 7.12	• 1 week to 3 months post-stroke<br>• 3.0 T<br>• 8 min<br>• ReHo measures, ALFF, BEN	• NIHSS, FMA, MMSE	• Compared to healthy controls, stroke patients presented significantly decreased BEN values in the contralesional precentral gyrus, bilateral dorsolateral superior frontal gyrus and bilateral SMA.<br>• There was a significant negative correlation between BEN and ReHo, as well as between BEN and ALFF in the contralesional precentral gyrus.<br>• Across all stroke patients, there was a significant positive correlation between FMA scores and mean BEN values in the ipsilesional SMA and ipsilesional dorsolateral superior frontal gyrus.
Bonkhoff, (2020) [20]	• First-ever ischemic stroke<br>• Cross-sectional<br>• 31 patients<br>○ 68.40<br>• 17 controls<br>○ 65.40	• 7.20 ± 2.70 days post-stroke<br>• 3.0 T<br>• 7 min<br>• ICA, static and dynamic functional network connectivity analysis, cluster analysis of dynamic functional network connectivity matrices, domain-wide segregation for static and dynamic connectivity configurations<br>• Lesion analysis	• NIHSS, ARAT	• The dynamic connectivity patterns of stroke patients for the identified motor networks (cortical, subcortical, cerebellar) diverged from those of healthy controls depending on the severity of the initial motor impairment<br>• Moderately affected patients spend significantly more time in a weakly connected configuration characterized by low levels of connectivity, both locally as well as between distant regions.<br>• Severely affected patients showed a significant preference for transitions into a spatially segregated connectivity configuration which featured particularly high levels of local connectivity within the three regional domains as well as anti-correlated connectivity between distant network across domains.<br>• A third connectivity configuration represented an intermediate connectivity pattern compared to the preceding two, and predominantly encompassed decreased interhemispheric connectivity between cortical motor networks independent of individual deficit severity.
Bonkhoff, (2021) [21]	• Acute ischemic stroke<br>• Cross-sectional<br>• 41 patients<br>○ 67.30 ± 9.90<br>• 0 controls	• 2–5 days post-stroke<br>• 1.5 T<br>• 6 min<br>• ICA, dynamic functional network connectivity analysis<br>• WMH analysis	• NIHSS	• There were three distinct dynamic connectivity configurations acutely post-stroke.<br>• State 1 was the most segregated state and appeared the least often, characterized by highly positive intra-domain connectivity, whereas highly positive inter-domain connectivity patterns found

				between cortical SMN and cerebellar as well as the visual domains.<br>• State 2 was the most frequent connectivity state, characterized by mostly neutral inter-domain and only slightly positive intra-domain connectivity.<br>• State 3 displayed high positive intra-domain connectivity in the SMN and visual domains (as State 1) and positive inter-domain connectivity between these domains.<br>• More severely affected patients spent significantly more time in a configuration that was characterized by particularly strong connectivity and isolated processing of functional brain domains<br>• Configuration-specific time estimates possessed predictive capacity of stroke severity in addition to the one of clinical measures.<br>• Recovery (change of the NIHSS over time) was significantly associated to the dynamic connectivity between intraparietal lobule and left angular gyrus.
Section B: Motor outcome following specific rehabilitation interventions and rs-fMRI analysis				
Fan, (2015) [22]	• Ischemic or hemorrhagic stroke<br>• Longitudinal<br>• 10 patients<br>○ 52.70 ± 6.50<br>• 0 controls<br>• Intervention: 4-week RBAT	• 46.8 ± 20.11 days post-stroke<br>• 3.0 T<br>• 6 min 10 s<br>• ROI-based rs-FC analysis (ROIs: ipsilesional and contralesional M1), whole-brain voxel-by-voxel analysis<br>• n/a	• FMA, FIM, WMFT-FAS	• After RBAT, motor performance, functional use of the affected arm and daily function improved in all participants.<br>• Reduced interhemispheric rs-FC between the ipsilesional and contralesional M1 (M1-M1) and the contralesional-lateralized connections were found before treatment.<br>• Participants showed improved interhemispheric M1-M1 connection during RBAT compared to baseline, probably illustrating the significance of correct symmetry between the hemispheres for optimal performance.<br>• Greater M1-M1 rs-FC after RBAT were associated with better motor and functional improvements.<br>• The pre-to-post difference in M1-M1 rs-FC was a significant mediator for the relationship between motor and functional recovery.<br>• Interhemispheric FC in the motor cortex may be a neurobiological marker for recovery after stroke rehabilitation.
Zhang, (2016) [23]	• First-ever stroke with upper extremities motor deficits at chronic phase<br>• Longitudinal<br>• 17 patients<br>○ 57.0 ± 8.55<br>• 15 controls	• 59.19 ± 15.152 days post-stroke<br>• 3.0 T<br>• n/a<br>• Seed-to-whole brain with ROI in the ipsilesional M1<br>• n/a	• FMA	• Compared to healthy controls, the FC in stroke patients was significantly increased between the ipsilesional M1 and the ipsilesional inferior parietal cortex, frontal gyrus, SMA, and contralesional angular and decreased between the ipsilesional M1 and bilateral M1.

	o 63.4 ± 8.11<br>• Intervention: motor imagery			• After motor imagery intervention, the FC between the ipsilesional M1 and contralesional M1 increased while the FC between the ipsilesional M1 and ipsilesional SMA and paracentral lobule decreased.<br>• A statistically significant correlation was found between the FC change in the bilateral M1 and the FMA score change.
Li, (2017) [24]	• Unilateral cerebral subcortex lesion in the MCA territory<br>• Longitudinal<br>• 12 patients<br>o 55.3<br>• 0 controls<br>• Intervention: 10-day rTMS	• Within 1-week post-stroke<br>• 3.0 T<br>• n/a<br>• Seed-to-whole brain with ROIs in ipsilesional M1<br>• Lesion analysis	• NIHSS, FMA, BI	• The rTMS group showed motor recovery while there was no significant difference of motor functional scores in the sham group before and after the sham rTMS.<br>• Compared with the sham, the rTMS treatment group showed increased FC between ipsilesional M1 and contralesional M1, SMA, bilateral thalamus, and contralesional postcentral gyrus.<br>• Decreased FC was found between ipsilesional M1 and ipsilesional M1, postcentral gyrus and inferior and middle frontal gyrus.<br>• Increased or decreased FC detected is an important finding to understand the mechanism of brain functional reorganization following rTMS.
Lefebvre, (2017) [25]	• Chronic stroke at least 6 months post-stroke<br>• Longitudinal<br>• 22 patients<br>o n/a<br>• Intervention: dual-tDCS during motor skill learning	• Baseline and after 2 weeks, following the dual-tDCS/sham or dual-tDCS during motor skill learning intervention<br>• 3.0 T<br>• 6 min<br>• ICA, ROI-to-ROI analysis within the motor/premotor network<br>• n/a	• Neurological examination	• In comparison to sham, the use of dual-tDCS and a single session of motor skill acquisition enhanced FC in chronic hemiparetic stroke patients for at least a week.<br>• Dual-tDCS increased FC in the bilateral somatomotor network, particularly in PMddamH, and in M1damH and PMddamH.<br>• A single session of tDCS has never been shown to have such a long-lasting impact on rs brain activity.<br>• The DMN, DAN, VN, and salience network rs-FC did not alter between the three sessions, but only the somatomotor network FC rose beyond baseline following the combination of dual tDCS and motor skill learning.<br>• The combined intervention may have altered the somatomotor network spontaneous activity for at least a week based on the network-specific modulation of FC.<br>• Based on ROI-to-ROI analysis, the ROI combination with the highest FC at baseline and one week after sham was M1undamH - PMdundamH. The highest FC was discovered between M1damH and PMddamH one week after combined dual-tDCS/motor skill learning.

				• The findings point to a reorganization of FC one week following dual-tDCS / motor skill learning.
Li, (2017) [26]	• First-ever subcortical ischemic stroke with unilateral motor deficits<br>• Longitudinal<br>• 17 patients<br>○ 63.29 ± 12.27<br>• 14 controls<br>○ 62.21 ± 10.48<br>• Intervention: Conventional Western medicine treatment, Acupuncture	• At least 3 weeks between stroke and study enrollment; Baseline and 1-month following clinical treatment<br>• 3.0 T<br>• 6 min<br>• Seed-to-whole brain with ROIs belonging to the motor execution network<br>• n/a	• Chinese Stroke Scale for Clinical Neurological Deficits	• Compared with the conventional Western medicine treatment group, the acupuncture combining conventional Western medicine group showed a significant enhancement of the percent changes of Chinese Stroke Scale for Clinical Neurological Deficits from pre- to post- treatment intervention.<br>• All patients showed significant changes of FC between the pair of cortical motor-related regions.<br>• After treatment, both patient groups showed a recovery of brain FC to the nearly normal level compared with the controls in these pairs.<br>• A significant correlation between the percent of changes of Chinese Stroke Scale for Clinical Neurological Deficits and the pre-treatment FC values of bilateral M1 in all patients was found.<br>• The FC strengths between bilateral M1 of stroke patients can predict stroke patients' treatment outcome after rehabilitation therapy.
Guo, (2021) [27]	• Subcortical ischemic stroke<br>• Longitudinal<br>• 33 patients<br>○ 64.48<br>• 0 controls<br>• Intervention: rTMS	• 1 week post-stroke<br>• 1.5 T<br>• n/a<br>• ICA and FC of the intramotor network ROIs<br>• n/a	• NIHSS, FMA, BI	• While low frequency rTMS predominantly concentrated on the contralesional hemisphere, high frequency rTMS significantly enhanced the functional connectivity of the motor network in the ipsilesional hemisphere.<br>• During the motor recovery with high frequency rTMS therapy, the interaction between bilateral SMA and ipsilesional M1 and contralesional PMA may be more important.<br>• The functional restoration and reorganization that occur after high frequency rTMS and low frequency rTMS, respectively, within the motor network, may both be responsible for the motor recovery.
Chen, (2022) [28]	• Acute ischemic stroke<br>• Longitudinal<br>• 63 patients<br>○ 57.40<br>• Intervention: rTMS	• 1-week post-stroke<br>• 3.0 T<br>• n/a<br>• ROI-to-ROI analysis using AAL, correlation network of whole-brain regions<br>• n/a	• NIHSS, FMA, ADL, MMSE, HAMD	• Changes in FC between intra- and inter-hemispheric motor networks were linked to the therapeutic effects of paired inhibitory-facilitatory rTMS on motor dysfunction.<br>• For early-stage cerebral stroke patients receiving coupled rTMS, FC alterations were substantially linked with the restoration of motor function.<br>• Following an early-stage ischemic stroke, paired inhibitory-facilitatory rTMS treatment is a potential approach for adjuvant rehabilitation therapy.
Section C: Motor outcome in association with functional connectivity and structural connectivity				

Chen, (2013) [29]	• First-ever ischemic stroke<br>• Cross-sectional<br>• 11 patients<br>○ 57.54 ± 9.08<br>• 11 controls<br>○ 59.06 ± 9.15	• 4–6 months post-stroke<br>• 3.0 T<br>• n/a<br>• seed-to-whole brain with ROIs in L and R M1<br>• Lesion analysis, DTI	• UE-FMA	• Substantial correlation between the WM integrity in the transcallosal M1-M1 tract and the UE-FM score as well as rs-FC between the L and R M1.<br>• No discernible association between WM integrity in the transcallosal M1-M1 tract and rs interhemispheric M1 FC.<br>• Interhemispheric motor cortex connection can be a measure of stroke motor dysfunction, but each has some independence from the other.
Kalinosky, (2017) [30]	• Chronic stroke<br>• Cross-sectional<br>• 10 patients<br>○ 66.70 ± 9.94<br>• 9 controls<br>○ 64.20 ± 7.73	• >6 months post-stroke<br>• n/a<br>• 6 min<br>• ICA, iSFC, nSFCor maps<br>• Lesion analysis, DTI	• FMA, Box and Blocks Test of Manual Dexterity, Wolf Motor Function Test	• Multiple brain areas in stroke subjects with significantly lower iSFC compared to controls (e.g. cerebellum, midbrain, thalamus).<br>• Association between cerebellar iSFC and hand motor performance.<br>• Decreased iSFC between regions connected by longer fiber pathways in stroke patients, specifically in areas associated with integrative cortical network nodes (such as cerebellum and prefrontal cortex).<br>• The findings highlight the significance of residual structural connection in cortical networks by showing that alterations following a stroke in both intrinsic and network-based structural functional correlations at rest are linked to motor function.
Lin, (2018) [31]	• First-ever ischemic or hemorrhagic stroke<br>• Longitudinal<br>• 31 patients<br>○ 52.8<br>• 20 controls<br>○ n/a	• Baseline, month 3 and 12 post-stroke<br>• 3.0 T<br>• 10.25 min<br>• ROI-to-ROI analysis between L M1 and R M1 with seeds for arm/elbow and hand/finger<br>• Lesion analysis, DTI	• NIHSS, Neurobehavioral assessment of motor, visual and cognitive functions.	• FA of the ipsilesional CST improved significantly from 3 to 12 months and was strongly correlated with motor performance.<br>• FA improved even in the absence of direct damage to the CST.<br>• Inter-hemispheric FC improved over time but did not correlate with motor performance at 12 months.<br>• Clinical variables (early motor score, education level, and age) predicted 80.4% of the variation of motor outcome, and FA increased the predictability to 84.6%.<br>• FC did not contribute to the prediction of motor outcome.
Lee, (2019) [32]	• First-ever unilateral supratentorial stroke at chronic phase<br>• Cross-sectional<br>• 24 patients<br>○ Mild, n = 11: 69.70<br>○ Severe, n = 13: 74.5<br>• 0 controls	• Mild: 95.7 days post-stroke, Severe: 119 days post-stroke<br>• 3.0 T<br>• n/a<br>• ICA<br>• Lesion analysis, DTI	• FMA	• No significant differences in intra-network FC between groups.<br>• Increased inter-network FC in the mild group for SMA-M1 in the affected hemisphere and SMA-DLPFC in the unaffected hemisphere and for lesion-M1 in the unaffected hemisphere compared with the severe group.<br>• No significant changes in FC in the severe group.<br>• FA values of motor-related regions in the affected hemisphere were higher in

				mild motor impairment than in severe motor impairment group.
Lu, (2019) [33]	• First-ever unilateral internal capsule stroke within the past 7 days and with motor deficits<br>• Longitudinal<br>• 17 patients<br>○ 52.18 ± 9.93<br>• 17 controls<br>○ n/a	• Day 7, 30, and 90 post-stroke<br>• 3.0 T<br>• n/a<br>• ROI-to-ROI analysis using SMN ROIs<br>• DTI	• NIHSS, FMA, BI	• At day 7 and compared to healthy controls, stroke patients exhibited significantly increased intranetwork FC in two R hemispheric regions (PreCG, paracentral lobule) and decreased intranetwork FC in three L hemispheric regions (paracentral lobule, precuneus, middle cingulum).<br>• Compared to the baseline, the interhemispheric FC between ROIs was decreased at day 7, then gradually increased from day 7 to 90 and returned to normal at day 90.<br>• From day 7 to day 90 post-stroke, FA in the bilateral (ipsilesional, contralesional) CST increased as well, NHISS scores decreased while FMA and BI progressively increased.<br>• The increased interhemispheric FC was positively correlated with increased BI after unilateral internal capsule stroke.<br>• Changes in interhemispheric FC were positively correlated with changes in FA for all ROIs in the contralesional CST but positively correlated with FA changes in the ipsilesional CST only for the ROIs in the centrum semiovale.
Xia, (2021) [34]	• Ischemic stroke<br>• Longitudinal<br>• 34 patients<br>○ 63.40<br>• 34 controls<br>○ 61.20	• Weeks 1,4, and 12 post-stroke<br>• 3.0 T<br>• 6 min 20 s<br>• Seed-to-whole brain with ROIs along the L and R M1<br>• Lesion analysis, DTI	• NIHSS, UE-FMA, BI, MMSE	• Interhemispheric FC restoration mostly occurred within 4 weeks post-stroke.<br>• Increased interhemispheric FC and ipsilesional CST-FA were significantly correlated with greater motor recovery from week 1 to week 4 post-stroke (multivariate linear regression analysis).<br>• Only increased FA of ipsilateral CST was significantly correlated with greater motor recovery during week 4 to week 12 post-stroke compared to interhemispheric FC.<br>• Dynamic structural and functional reorganizations following motor stroke, and structural reorganization may be more related to motor recovery at the late subacute phase.
		Section D: Sensory outcome and rs-fMRI analysis		
Bannister, (2015) [35]	• First-ever infarct<br>• Cross-sectional<br>• 10 patients<br>○ 58.69<br>• 10 controls<br>○ 60.60	• Month 1 and 6 post-stroke<br>• 3.0 T<br>• 5 min<br>• Seed-to-whole brain with ROIs in L and R M1 and S1, as well as L and R somatosensory ventroposterior lateral thalami<br>• Lesion analysis	• NIHSS, ARAT, Wrist Position Sense Test, Functional Tactile Object Recognition Test, Rolyan® Hot and Cold Discrimination Kit, Tactile Discrimination Test, Weinstein Enhanced Sensory Test	• The bilateral S1 and motor (BA 4a, 6) regions exhibited considerable FC with the SI seeds for both hemispheres in healthy controls.<br>• At 1-month post-stroke, the stroke group showed deficient interhemispheric FC for both SI seeds, with each S1 seed functionally associated exclusively with nearby motor and SI areas.

				• Interhemispheric SI connection appeared to be returning for the stroke group at 6 months post-stroke.<br>• Contralesional S1 and a cluster located in the contralesional cerebellum and hippocampus had considerably higher FC at 1-month post-stroke than at 6 months.<br>• Contralesional thalamus and a cluster in ipsilesional middle cingulate cortex had significantly improved FC at 6 months.<br>• Interhemispheric FC associated with the sensori-motor network is impaired 1-month post-stroke and begins to improve toward normative levels 6 months later.
Goodin, (2018) [36]	• First-ever ischemic or haemorrhagic stroke with somatosensory upper limb involvement<br>• Cross-sectional<br>• 28 patients<br>o L lesions: 49.00 ± 12.04<br>o R lesions: 49.29 ± 13.55<br>• 14 controls<br>o 50.93 ± 16.26	• At least 3 months post-stroke<br>• 3.0 T<br>• n/a<br>• Seed-to-whole brain with ROIs in L and R S1 and S2, laterality index of FC<br>• Lesion analysis	• NIHSS, Tactile Discrimination Test, Weinstein Enhanced Sensory Test, Visual Analogue Scale	• Inter-hemispheric FC was greater in healthy controls compared to the combined stroke cohort from the L S1 seed and bilateral S2 seeds.<br>• The L lesion subgroup showed decreased FC compared to healthy controls, from the L ipsilesional S1 to contralesional S1 and to distributed temporal, occipital, and parietal regions.<br>• The R lesion group showed decreased FC from contralesional L S1 and bilateral S2 to ipsilesional parietal operculum, and to occipital and temporal regions.<br>• The R lesion group also showed increased intra-hemispheric FC from ipsilesional R S1 to inferior parietal regions compared to controls.<br>• Compared to the L lesion group, the R lesion group showed greater intra-hemispheric FC from L S1 to L parietal and occipital regions and from R S1 to R angular and parietal regions.<br>• Laterality Indices were significantly greater for stroke subgroups related to healthy controls for contralesional S1 (L lesion group) and contralesional S2 (both groups).<br>• The study highlights the influence of hemisphere of lesion on differences in location and laterality of functional connectivity alterations in stroke survivors with impaired touch sensation.<br>• Functional connectivity changes were found both within the somatosensory functional network and in interactions with other, associated functional networks.

Notes. rs-fMRI = resting-state functional magnetic resonance imaging; PPH = partially paralyzed hand; CPH = completed paralyzed hand; ROI = region of interest; FMA = Fugl-Meyer Assessment ; PHFA = Paralyzed Hand Function Assessment; MMSE = Mini-Mental State Examination; SMA = supplementary motor area; MCA = middle cerebral artery; FC = functional connectivity; DMN = default mode network; L = left; R = right; ARAT = Action Research Arm Test ; CST = corticospinal

tract; M1 = primary motor cortex; rs-FC = resting-state functional connectivity; SMN = sensorimotor network; NIHSS = National Institutes of Health Stroke Scale; mRS = modified Rankin Scale; i = ipsilesional; c = contralesional; ICA = independent component analysis; ALFF = amplitude of low frequency fluctuations; PMC = primary motor cortex; ReHo = regional homogeneity; BEN = brain entropy; WMH = white matter hyperintensities; RBAT = robot-assisted bilateral arm therapy; FIM = functional independence measure; WMFT = Wolf Motor Function Test; rTMS = repeated transcranial magnetic stimulation; BI = Barthel Index; tDCS = transcranial direct current stimulation; DAN = dorsal attention network; VN = visual network; ADL = activities of daily living; HAMD = Hamilton Depression Scale; UE-FMA = upper extremities Fugl-Meyer Assessment; DTI = diffusion tensor imaging; iSFC = intrinsic structural-functional connectivity; FA = fractional anisotropy; S1 = primary sensory area; S2 = secondary sensory area; n/a = non-applicable
